# 3D Printed Long-Range Cavity Structure UHF RFID Tag Antenna with Painting Conductive Ink on Convex Surface

**DOI:** 10.3390/s21041408

**Published:** 2021-02-18

**Authors:** Franck Kimetya Byondi, Youchung Chung

**Affiliations:** Information and Communication Engineering Department, Daegu University, Kyungsan 38453, Korea; kimetyafrank@gmail.com

**Keywords:** RFID tag antenna, long-range RFID tag, cavity antenna, convex tag antenna, RFID metal tag, RFID sensors, painted RFID tag antenna, IoT RFID sensor, 3D antenna, concave antenna, energy harvesting

## Abstract

In this paper, we describe a long-range convex cavity-type passive ultra-high-frequency (UHF) radio frequency identification (RFID) tag to use on various metal and non-metal surfaces, for IoT sensor energy harvesting. The tag antenna is built on the 3D printed cavity structure with polylactic acid (PLA) plastic and painted with the conductive ink on the 1 mm protruding area (convex) of inner surface and the side-walls of the cavity structure to form a cavity structure. The tag is designed to operate in the UHF band (840–960 MHz). This long-range cavity tag antenna (CTA) works at both 920 MHz and 915 MHz UHF RFID frequencies. It provides a linear polarized (LP) frontal reading range of 35 m and side reading range above 15 m when mounted on either metal or non-metal objects. We describe the antenna characteristics, structure, modeling, simulation, and experimental results. A mathematical reading range also was calculated and compared with experimental data.

## 1. Introduction

Radio frequency identification (RFID) uses a backscattered electromagnetic field to identify tagged objects or people, and to date many applications and systems have been introduced [1]. In an RFID system, an interrogator sends the electromagnetic field to power the tag antenna attached to an object, and then the tag antenna uses that power to send back the object data requested by the interrogator. There are passive RIFD tag antennas without a battery having a short reading range of 5–15 m [2,3], while active 433 MHz RFID tag antennas with a battery having a reading range over 1 km [4,5,6]. RFID uses different frequencies according to the applications. There are low-frequency (LF), high-frequency (HF) and ultra-high-frequency (UHF) RFID tag antenna [7]. RFID applications are numerous, such as tagging animals, asset tracking, electronic passports, smart cards, shop security, health care, transport management system, and object logistics [8].

In automotive and other industry applications, tagging a metal object is of strong interest [9], and a special design for the passive tag to attach on metal without interference is required [10]. There are many papers published about UHF RFID passive tags to attach to metal object. Some are: a small-sized dual-band tag for metal objects is developed in reference [11]. This presents a long reading range of 12 m and can be used in applications like automotive, aircraft and energy industry. A 900 MHz UHF RFID metal printed inverted F antenna (PIFA) tag antenna is designed in [12] for the identification of items that are used in the electricity distribution network, which has a hostile operating environment to default the normal use of RFID. The antenna was designed to provide a 16 m reading range, regardless of when it is placed in hostile electricity installation. A simulation study-based half-wave planar antenna with a parasitic element for a metal-mountable UHF RFID tag is in work [13]. The bandwidth in the reading range of this tag antenna can be extended by increasing the electrical length of the parasitic element. The simulation result reading range reached 18 m. The authors in [14] developed a long-range metal mountable patch-type tag antenna for passive UHF RFID systems. The tag antenna has a total height of 3.3 mm and a 25 m theoretical line-of-sight read range on metal. The overall footprint design with the substrate and ground plane was 68 × 131 mm. Paper [15] introduces a single ID UHF RFID tag antenna for both long-range applications like car parking, and a short range for application like office door control.

A metal cavity type UHF RFID tag antenna is presented in reference [16] with a 219 × 229 × 88 mm^3^ cube shape and the measured reading range is 8 m. The design allows a dipole tag antenna to be read in the metal environment. Reference [17] presents the longest reading range cavity type UHF RFID tag antenna with a 26 m measured reading range and 36 m mathematical calculated reading range. This compares the performance of other tags’ antennas in a large table and proves that the tag has a longer reading range than previously published numerous long-range RFID tag antennas for metal and non-metal applications. Furthermore, the paper with its longest reading range can be implemented in the IoT sensor network for energy harvesting in long distances by replacing the tag chip with the energy harvesting circuits.

This paper introduces a new method to design an antenna. The antenna structure is designed and fabricated with 3D printed convex surface (1 mm protrude surface) antenna parts. Instead of designing an antenna with the conductive material, the proposed antenna is designed by painting conductive ink on the convex surface of the antenna instead of the flat surface of the antenna. For fabricating an antenna on 3D printed plastic, generally, the designer uses conductive tape or painting with conductive paint. The 3D printed surface is not smooth enough to attach the tape, and not easy to attach the conductive tape to the right place to fabricate a delicate antenna design. In addition, painting conductive ink on an object is not a proper method to design and fabricate an antenna since the liquid conductive ink smears out, therefore it is impossible to accurately paint the ink at the edges of the antenna. 

3D polylactic acid (PLA)-printed convex cavity type UHF RFID tag antenna for a non-metal and metal environment has been designed and fabricated. The conductivity of conductive ink has been tested based on the different thickness of conductive painting. The concave-type tag antenna and convex were simulated and fabricated with PLA plastic. The convex type is easier to paint and fabricate than the concave surface. Therefore, the 3D printed with a convex surface and conductive ink painted cavity structure UHF RFID tag antenna was designed for metal and non-metal environments with a measured read range of 35 m.

The structure of this paper is organized as follows. Section 2 presents the needs of the 3D convex cavity tag antenna structure, Section 3 presents our proposed convex long-range UHF tag antenna with a detailed design process. Finally, Section 4 concludes our work. 

## 2. Benefit of 3D Cavity Concave–Convex Antenna

**Figure 1:**
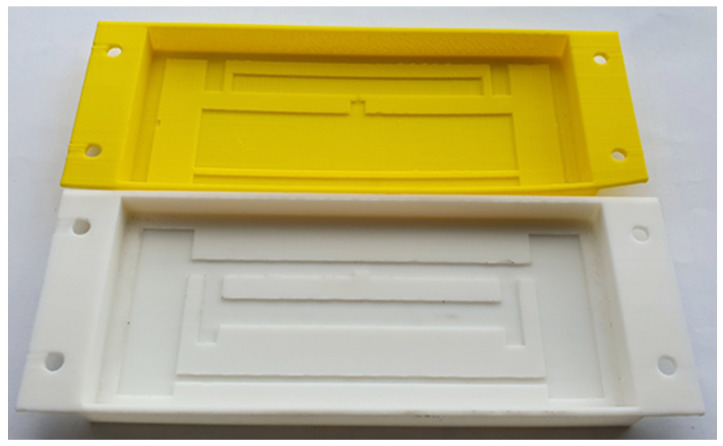
Yellow PLA convex and white ABS concave 3D cavity antenna structure.

Designing and manufacturing an antenna using a 3D printer presents several benefits such as the 3D shape, which can play the role of the high-gain antenna. The 3D printing allows us to fabricate antennas accurately and easily, and it also allows us to change the antenna structure easily. The 3D printed cavity structures with concave and convex structures of antenna were designed and are shown in Figure 1. The convex (1 mm protrude surface) surface structure is fabricated with yellow PLA, and the concave (1 mm recessed) type antenna is fabricated with white acrylonitrile butadiene styrene (ABS) plastic shown in Figure 1. The regions or areas of conductive parts of antennas are painted with conductive ink, called the convex or concave surface of the inner surface of the cavity structures shown in Figure 1. The height of the concave area is lower than the flat surface, and the height of the convex area is higher than the 3D printed flat surface of the plastic shown in Figure 1.

PLA is more biodegradable than ABS, and from renewable resources such as corn starch or sugarcane. It is somewhat the most popular bioplastic, used for plastic cups and medical implants. Therefore, PLA has been used to fabricate the convex antenna structures.

A cavity structure antenna in [17] explains that putting a simple tag antenna directly attached to a metal object causes a loss of the most important characteristic of a passive tag—the reading range. An antenna cannot work when directly placed on metals or high permittivity [18]. This is because the metal object in the presence of the electromagnetic field enables the loss and change of impedance of the tag antenna. To solve the problem, a dielectric gap must be inserted between the tag antenna and the metal object. Among the dielectric used, there is Styrofoam or air. The metal object represents the bottom of the cavity, and the four sides of the dielectric wall must be covered with metal to form the cavity shape.

The proposed cavity type 3D printed UHF tag antenna in this article is painted with Elcoat silver paste on the convex area of the inner surface of the cavity structure antenna. The four inner sides of the cavity structure have also been painted with conductive ink to form the cavity antenna structure. The metal object, where the PLA 3D plastic is attached, forms the back metal of the cavity structure. Since the PLA 3D structure has on its inner side the shape of the antenna, the Elcoat silver paste is painted to the shape of the antenna. The inner side of the cavity antenna is empty air. The air gap isolates the tag antenna to touch the back metal on which the convex cavity PLA is attached. This convex tag antenna–air–metal cavity structure characteristic results in a long reading range of our design. When attaching the convex tag antenna to a non-metal object, the back-side copper metal needs to be added to keep the cavity structure performance. The location of the printed antenna is the inner side of the 3D printed cavity structure. The 3D plastic cavity protects the antenna and forms the cavity structure. When attaching the convex tag antenna to the metal object, this metal object will play the role of back metal to realize the cavity structure. We designed and fabricated the final antenna with the convex shape surface since it outperforms the concave surface cavity antenna.

## 3. Convex RFID Tag Antenna Design

This antenna design will help the engineer understand the antenna theory background and put it into practice by designing a UHF RFID sensor tag for the IoT system step by step. The basics of UHF wave propagation enumerated here will give an idea of the physical characteristics of UHF waves and help to design an antenna fitting with the proper application. Antenna parameters that impact antenna performance will be shown. The antenna simulation will be performed by using computer simulation technology (CST) [19] and fabricated with a 3D printer. Antenna tuning capabilities will be shown too.

### 3.1. UHF RFID Tag Antenna Wave Propagation and Reading Range

**Figure 2:**
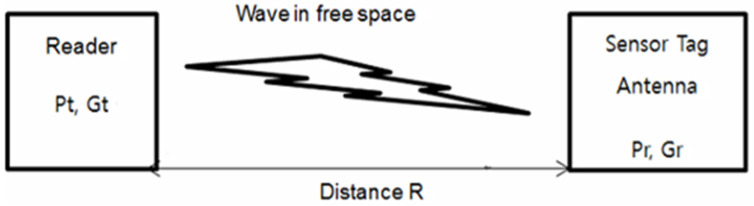
Propagation diagram in free space.

Figure 2 shows the communication in free space between the reader’s transmitting antenna and a sensor tag receiving antenna. The Friss Equation (1) [20,21,22] shows the relation between all parameters involved in the transmission and receiving system. Those parameters are transmitted power $P_{t}$, transmission antenna gain $G_{t}$, tag antenna received power $P_{r}$, gain of reader antenna $G_{r}$, wavelength *λ* and the distance between the transmitting and the receiving antenna *R*:

(1) $$P_{r}=P_{t}{\left(\frac{\lambda }{4\pi R}\right)}^{2}G_{t}G_{r}\;,$$

The maximum power delivered from the reader to the tag antenna is given if the tag antenna input impedance ($Z_{a}$) is a complex conjugate matching the transponder chip impedance ($Z_{c}$). Thus, $Z_{a}$ = $Z_{c}^{*}$, separated into real and imaginary, we have real part $R_{a}$ = $R_{c}$ and imaginary part $X_{a}$ = −$X_{c}$. Read range is the most important tag performance characteristic. It can be calculated with τ which is the matching coefficient that determines how well the tag antenna and IC chip are matched (2), and the reading range is calculated with Equations (2) and (3) [3,21,23]:

(2) $$\tau \;=\;\frac{4R_{a}^{2}}{|Z_{a}+Z_{c}{|}^{2}}$$

(3) $$\;RR=\;\frac{\lambda }{4\pi }\sqrt{\;\frac{P_{t}G_{t}G_{r}}{P_{th}}\;\tau }\;\;\;\;\;\;\;\;\;\;0\;\leq \;\tau \;\leq \;1,$$

### 3.2. UHF RFID Tag Antenna Design Flow

The simulation is performed using computer simulation technology (CST). We start by defining the constant values that are the dielectric constant of air (ε = 1), and the PLA plastic cover ε = 1.3, which were then inserted into the CST for design. The other constant is the tag sensitivity, which is also called the minimum operating power supply $P_{th}$ which is equal to −20.5 dBm, provided by the Alien Higgs 4 datasheet. Alien’s Higgs-4 chip operates in the frequency range of 840–960 MHz. Thus, $P_{th}$ (data sheet) can be expressed in watt as Equation (4):

(4) $$\;\;\;\;\;P_{th}=10^{\frac{-20.5}{10}}=8.913\;uW$$

#### 3.2.1. Convex Tag Antenna

**Figure 3:**
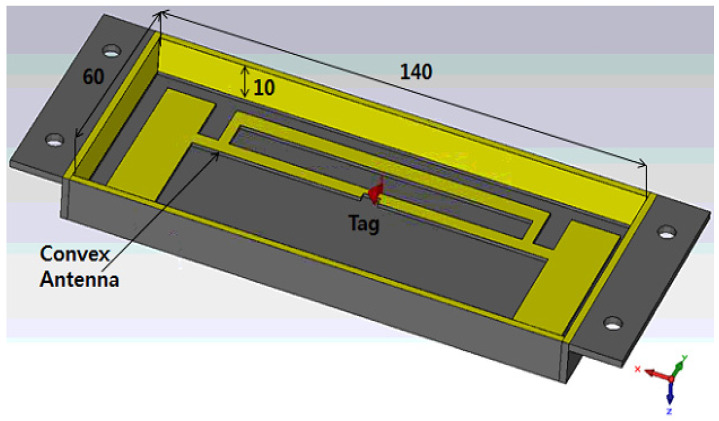
3D shape of the convex cavity structure tag antenna.

Figure 3 shows the inner shape of the convex tag antenna in yellow. The convex antenna is at 1 mm protruding with the top inner PLA cover which is called the convex area. The inner size of the cavity is 140 × 60 × 10 mm. The copper in yellow color is on four sides of the PLA plastic, and the copper on four edges allows contact with the metal to which the cavity will be attached (back copper). This copper metal helps to form the cavity structure. The concave (1 mm recessed) tag antenna has been built similarly to the convex antenna, however, the performance was not as good as that of the convex one because the liquid conductive ink clumps in the corner of the convex area of the antenna. Therefore, only the design of a convex structure is shown here.

**Figure 4:**
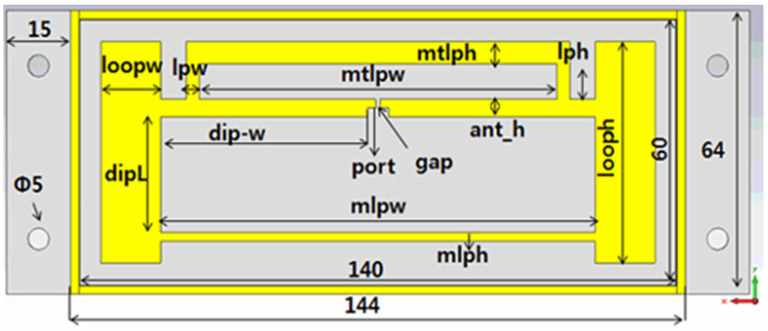
Parameters of the convex cavity structure tag antenna.

Figure 4 shows the 2D CST design of the convex tag antenna design. The antenna has two main parts. The dipole part of the antenna with length of dip-w, ant_h, dipL, port, and gap. The T-matching loop area of the antenna, which is the matching network with mtlpw, mtplh, lph, lpw as first matching parameters and the second matching loop parameters loopw, looph, mlph, and mlpw.

#### 3.2.2. Tuning of an Antenna

The loop size and the dipole length are the parameters to use for tuning the antenna to obtain proper matching between the antenna and chip impedance. Loop size and dipole size parameters are shown in Table 1. The parameter values in the table are the final values obtained after many tunes in CST simulation. The tuning is performed for ant_h which equals 4 mm, and ant_h which equals 9 mm. These two resonate at a different frequency.

**Figure 5:**
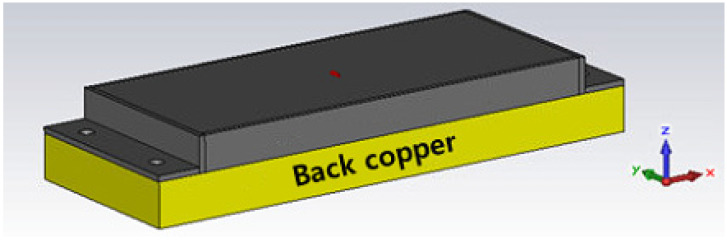
Shape of the antenna on the back copper material mimic attached on the metal object.

According to the simulation, the variation of the size of back copper mimic of the metal object in Figure 5 did not show a big change in the value of reflection coefficient S11. This can be interpreted as no matter the size of the metal where the convex antenna is attached, the antenna characteristic reading range will not be significantly changed. 

**Figure 6:**
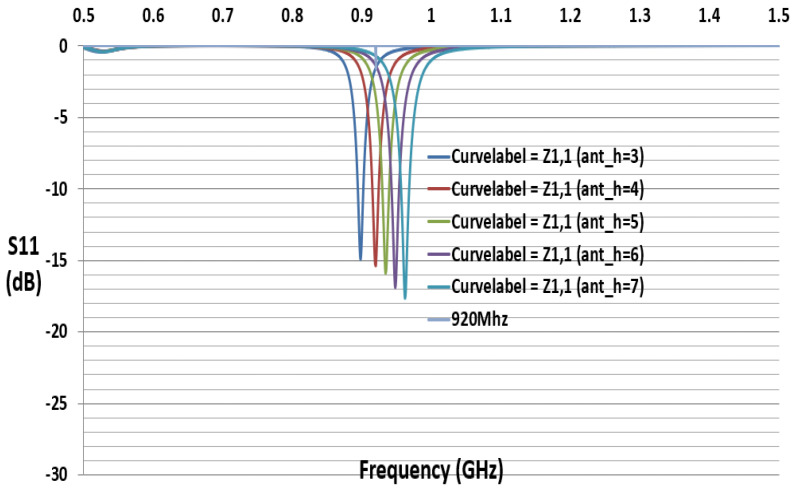
Simulated S11 results with variation of parameter ant_h (mm).

**Figure 7:**
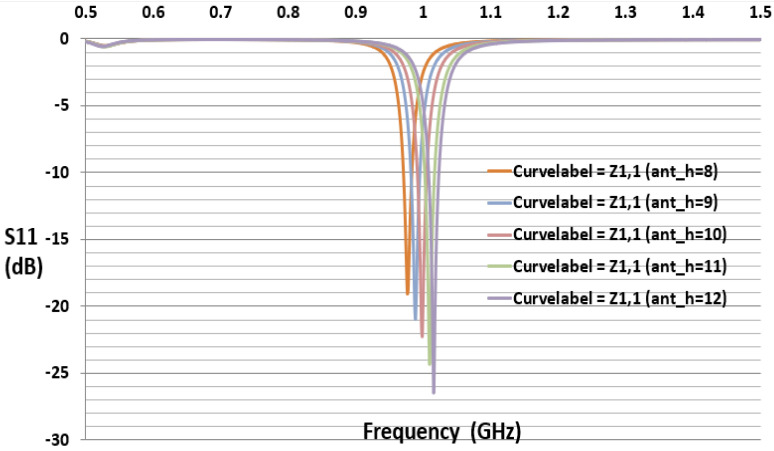
Simulated S11 results with the variation of parameter ant_h (mm).

The reflection coefficient describes either the amplitude or the intensity of a reflected wave relative to an incident wave and hence describes the ratio of the reflected wave to the amplitude of the incident wave. A low reflection coefficient is an indication of good matching between chip impedance and antenna impedance. Figure 6 and Figure 7 show the parameter sweeping simulation result of the reflection coefficient S11 by tuning the value of ant_h. The matching at the resonance frequency 920 or 988 MHz between the convex antenna and the Higgs4 chip impedance was calculated using S11 Equation (5). The result shows that the UHF convex tag antenna (CTA) resonates at 920 MHz while other parameters resonate at a higher or lower frequency than 920 Mhz. The chip impedance is calculated using the parallel impedance Equation (6):

(5) $$S11\;\left(\mathrm{dB}\right)=\;20\log_{10}\;\left(\frac{Z_{a}-\;Z_{c}^{*}}{Z_{a\;}+Z_{c}}\right)$$

(6) $$Z_{c}=\;\frac{R}{1+\;\omega^{2}R^{2}C^{2\;}}\;-\;j\frac{\omega R^{2}C}{1+\;\omega^{2}R^{2}C^{2}}\;$$

where $Z_{c}$ is the chip impedance, angular frequency ω=2πf, *R* is the chip resistance, and *C* is the chip capacitance (refer to the Alien Higgs-4 datasheet).

**Figure 8:**
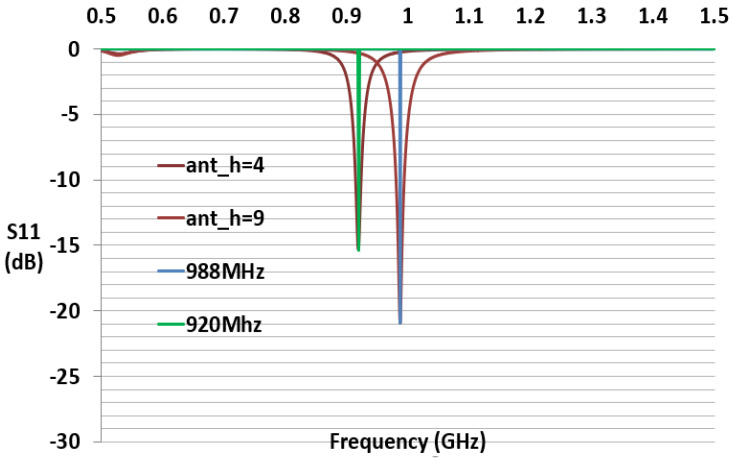
Simulated S11 with parameter ant_h = 4 and 9 mm.

Figure 6 and Figure 7 show that the simulation S11 value of the parameter ant_h varies from 3 to 12 mm, respectively, and S11 equals −15 dB at 920 MHz and S11 equals −21 dB at 988 MHz. Figure 8 shows only the results of S11 with ant_h = 4 mm, and ant_h = 9 mm cases, to compare them clearly. The simulation antenna impedance value is calculated using this complex equation $Z_{a}=a+jb$, where a is the real part and b is the imaginary value. At 920 MHz, the antenna impedance is $Z_{a}=12.98+j181.17$ and $Z_{c}$ is the chip impedance. In the Alien Higgs-4 datasheet, R=1800 kΩ and C=0.95pF. The frequency ω=2πf, where *f* is 920 MHz. Using Equation (6), the chip impedance is $Z_{c}=18.258-j180.34$ at 920 MHz. The conjugate chip impedance is $Z_{c}^{*}=18.258+j180.34$. 

**Figure 9:**
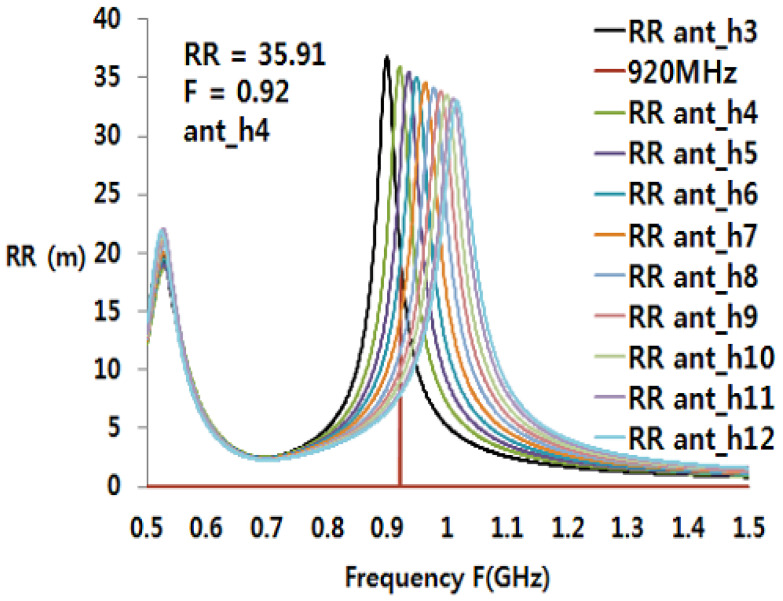
Simulation of the reading range by the variation of ant_h value (mm).

The impact of ant_h on the matching and performances is shown in Figure 9. Figure 9 shows the parametric reading range of the tag in the function of the ant_h dimension. It can be observed how the tuning of the antenna will change by increasing the loop dimension parameter ant_h. An increase in the loop dimension will decrease the resonance frequency (loop “resonance”) of the tag antenna. This means that if the ant_h increases, the loop area will decrease, and the resonating frequency increases. For ant_h equals 4, the reading range *RR* = 35.91 m. The reading range was calculated using the Equation (3) where τ is given by

(7) $$\tau =\;\frac{4R_{c}R_{a}}{{\left[Z_{c}+Z_{a}\right]}^{2}}=\;0.938,\;0\;\leq \;\tau \;\leq \;1,$$

$$\mathrm{Where},\;\;{\left[Z_{c}+Z_{a}\right]}^{2}={\left(R_{a}+R_{c}\right)}^{2}+{\left(X_{a}+X_{c}\right)}^{2}.$$

**Figure 10:**
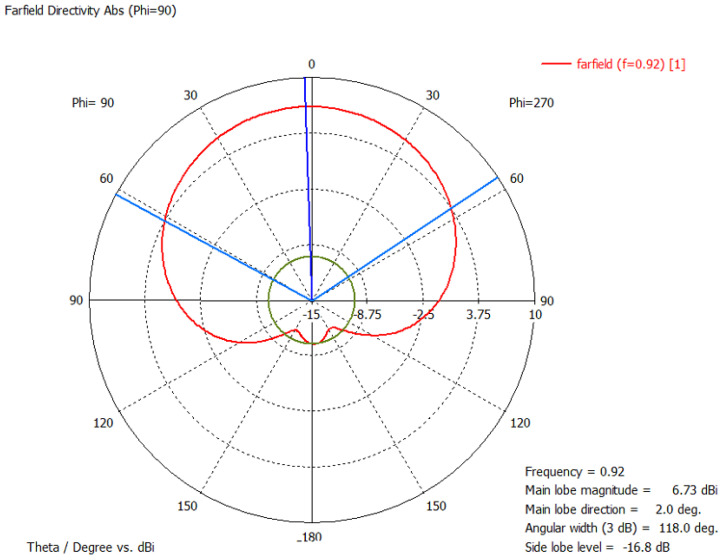
Simulated radiation pattern of YZ plane.

Note that $Z_{a}$, $R_{a}$, $X_{a}$ are CST antenna simulation data. The calculation here uses antenna simulation data for ant_h = 4 mm. $G_{r}$ is the tag antenna gain. The gain is 6.73 dBi as stated in the simulation result of the gain pattern in Figure 10. The gain can be written as

(8) $$G_{r}=\;10^{\frac{6.73}{10}}=4.71$$

The radiation pattern of the convex RFID tag antenna is shown in Figure 10. Due to the metal cavity, at 920 MHz, the gain of the antenna is 6.73 dB, the direction of the main lobe 2.0 degree (blue line), and the 3 dB angle in the main direction is 118 degree (sky blue line).

**Figure 11:**
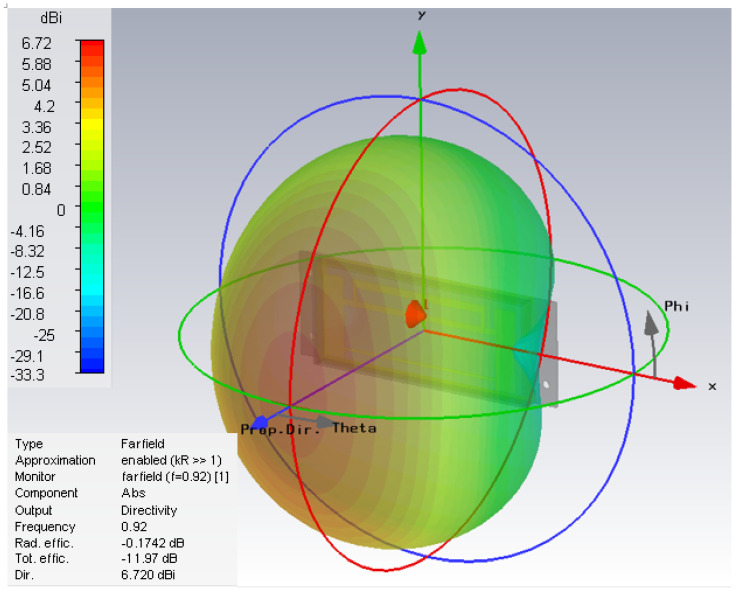
Simulated 3D radiation pattern.

Figure 11 shows the simulated 3-D convex tag antenna radiation pattern at 920 MHz. The convex tag antenna structure is positioned in the x–y plane with arms pointing in the z direction. The simulated 3-D characteristics of the label antenna show a typical dipole behavior and the directivity of maximal 6.72 dB in the x and in z direction. The 3D radiation pattern of the antenna, which would be seen by an ideal linear polarized reader antenna, moving around the y axis rotated with the angle Phi (the rotation angle around the z axis); and moving around the x axis rotated with the angle Theta (the angle in the x–z plane). 

#### 3.2.3. Fabrication and Result of Long-Range RFID Tag on Convex Surface

**Figure 12:**
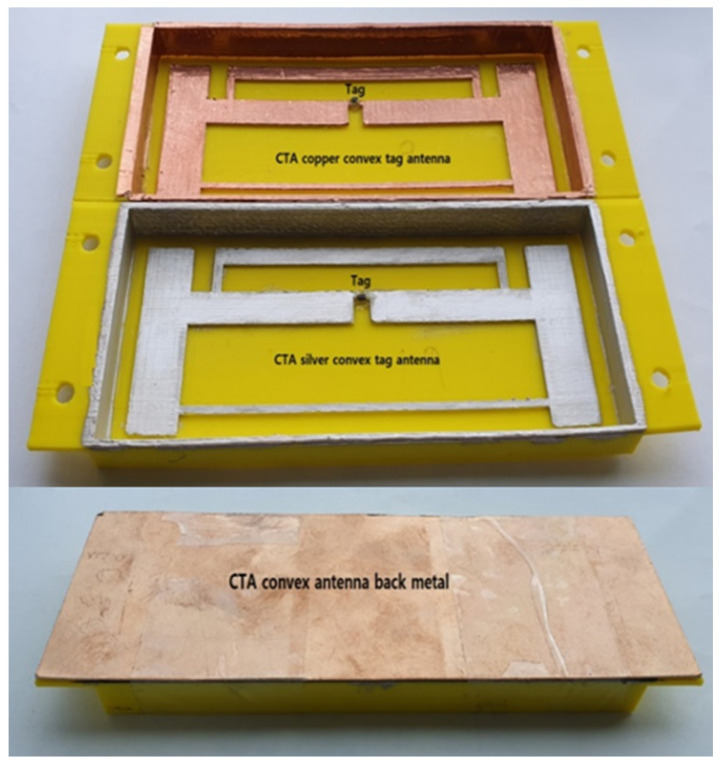
Fabricated copper and silver painted tag antenna (ant_h = 9 mm).

The Figure 12 shows the fabricated copper and painted silver CTA RFID tag antenna inner and back metal views for convex with ant_h equals 9, respectively. The fabricated figure for ant_h looks like Figure 1, the copper CTA, antenna, side copper, and back copper all are attached on yellow PLA plastic. For the silver CTA tag antenna, the silver paste is painted on a convex antenna shape and four sides of PLA plastic. The antenna was fabricated using copper and another SILVER COAT paste. The PLA plastic was fabricated using a 3D printer. The UHF RFID strap has been attached with the silver adhesive slow hardening solder.

We fabricated 12 convex cavity tag antennas with 12 different values of ant_h. Some of the different tag antennas had different sizes of ant_h. Table 2 shows the comparison of the simulation and outdoor measured reading range of multiple antennas, which differ by ant_h value and by fabrication material type (copper c, silver paste s). The parameter ant_h = 4 mm resonated at 920 MHz with the reflection coefficient of S11 −15 dB, and S11 equals −9.9 dB for 915 MHz. The reading range measured by Alien’s reader was 5–11 m for the two fabricated antennas, while the simulation calculated reading range was 35.91 m. The difference in result was caused by the fabrication loss which caused a mismatch between the tag antenna and the IC chip. This may be from the soldering resistance, less than 1 ohm.

Therefore, we tuned the antenna for high frequency. Antenna ant_h = 9 and 10 mm were also fabricated but simultaneously tuning to higher resonating frequencies of 988 and 998 MHz. The CTA ant_h9 had a reflection coefficient of S11 = −20.94 dB and was fabricated with copper and silver paste. The ant_h9 with copper had a measured reading range (RR) maximum of 35 m with a 920 MHz Alien reader. Silver ant_h9 was 36 m RR. The CTA ant_h10 also shows a similar result with ant_h9. Their simulation resonance was 988 and 998 MHz, respectively, but they presented a measured RR with the Alien reader 920 MHz of about 36 m. This result was also due to fabrication loss. However, this gives us a way of obtaining a long-range fabricated CTA tag antenna.

**Figure 13:**
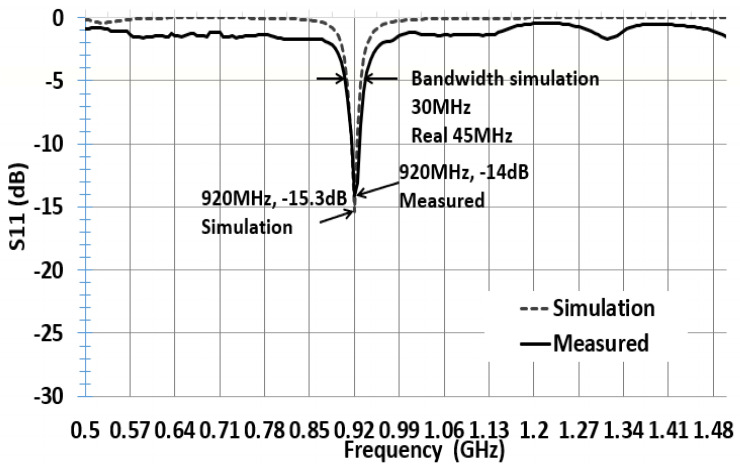
Simulated and measured S11 with parameter ant_h4 in Table 2.

**Figure 14:**
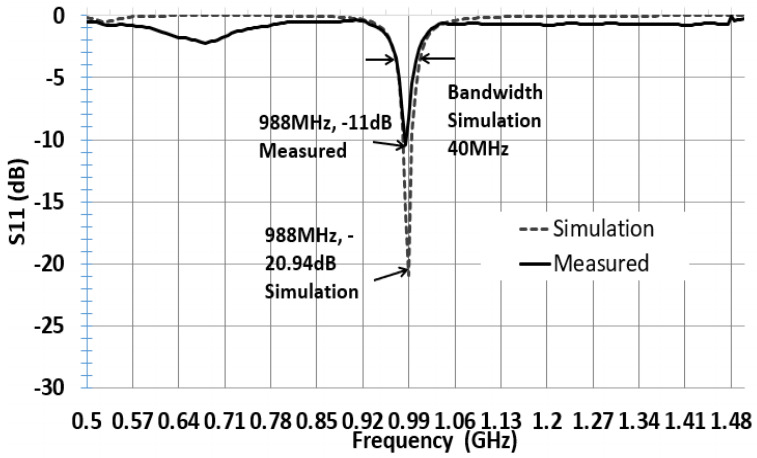
Simulated and measured S11 with parameter ant_h9 in Table 2.

Figure 13 shows the graph of simulated and fabricated ant_h4 CTA tag antenna. The simulation reflected that coefficient S11 is −15.3 dB. After the fabrication of the antenna, we measured the reflected coefficient using Agilent’s E5071B network analyzer. The antenna presents a measured S11 of −14 dB. The bandwidth at −3 dB is 45 MHz, the frequency range is from 897.5 to 945.5 MHz, considering 920 MHz as the center frequency. This shows that our antenna can operate at both 920 and 915 MHz according to the use. Figure 14 shows the graph of the simulated and fabricated ant_h9 which resonate 988 MHz, and the simulation reflected coefficient S11 is −20.94 dB with 40 MHz bandwidth. The measured reflected coefficient S11 is −11 dB.

**Figure 15:**
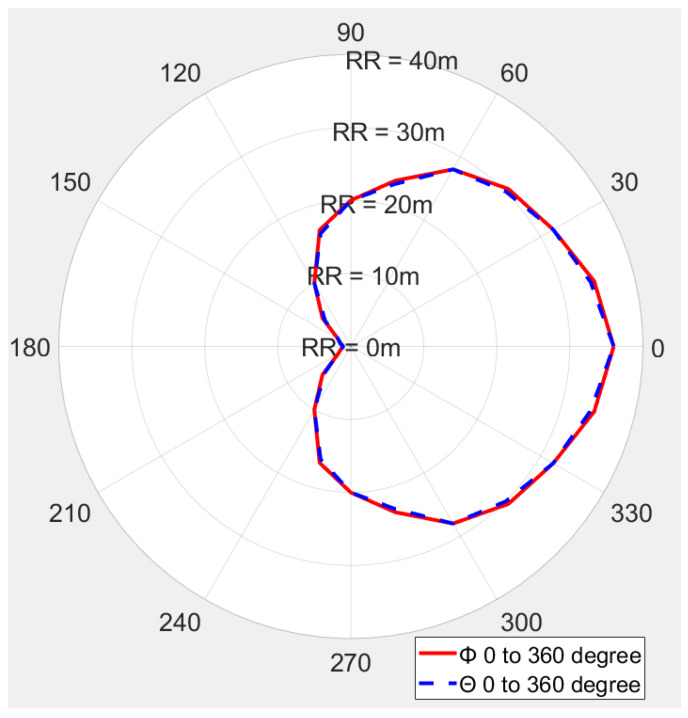
The reading range pattern of a fabricated long-range cavity tag antenna (CTA) ant_h9, CP reader antenna.

We measured the reading range with an Alien RFID reader with a linear polarized linear polarized (LP) antenna, and a circular polarized (CP) antenna. Figure 15 shows the outdoor reading range pattern of the fabricated CTA with ant_h9 size measured using CP antenna. The measurement was performed by turning the antenna according to Phi and Theta angle directions. The backside of the antenna where there is metal shows a short reading range of 1 m at 180 degrees, while the front side shows the maximum reading range of about 36 m at 0 degrees. The simulated radiation pattern also shows that the maximum power of the antenna is obtained when the convex tag antenna faces the reader.

The shape of the measured reading range pattern is circular like the simulated radiation pattern in Figure 10, when measuring the reading range with a CP antenna. The shape of the measured reading range pattern is flat instead of the circle when using an LP reader antenna.

## 4. Discussion

This work presents a convex UHF tag antenna with a long reading range and wideband range. This mathematically and experimentally demonstrates that our proposed (ant_h = 9 and ant_h10) long-range UHF RFID convex cavity-type tag antenna (CTA) has a longer reading range than the existing passive tag. 3D polylactic acid (PLA)-printed convex cavity type UHF RFID tag antenna for non-metal and metal environments has been designed and fabricated. Our tag was designed to be attached to the metal object and nonmetal object with 140 × 60 × 10 mm size and reached a 36 m measured reading range and 33 m simulation mathematically calculated reading range. It is longer than the existing passive tag antennas. The difference in results is based on the fabrication and misalignment problems. The back copper of our antenna can be replaced by the metal on which the antenna will be attached. The cavity structure used air instead of Styrofoam. There is no RF board; the tag antenna is attached on the top-inner side of the plastic, PLA cover. Thus, our structure saves material. In addition, with this long-range advantage, the proposed CTA tag antenna can be designed to harvest power for the IoT sensor network by replacing the tag chip with the energy-harvesting circuits.

## Figures and Tables

**Table 1:** Convex tag antenna parameters.

Parameters	Size (mm)	
	ant_h4	ant_h9
dip-w	48.5	48.5
**ant_h**	**4**	**9**
mlph	2	2
mlpw	101.5	101.5
looph	50	50
loopw	14	14
port	2	2
dipL	24.5	20
gap	1	1
lph	7.5	7.5
lpw	2.5	2.5
mtlpw	83.5	83.5
mtlph	5	5

**Table 2:** Simulation and measured parameter reading range with different sizes of ant_h.

Parameters	Simulation Data Based Calculated Reading Range (RR)							Outdoor Real Measured RR (m) with LP Antenna. Copper c, Silver s
	Resonance frequency F			S11 (dB)		RR(m) at 920 MHz	RR(m) at 915 MHz	

	F (GHz)	S11 (dB)	RR (m)	920 MHz	915 MHz			

ant_h3	0.899	−14.3	36.7	−1.57	−2.34	20.08	23.67	Not made
ant_h4	0.92	data	35.91	−15.3	−9.9	35.91	34.7	(s.2pc) 5–11
ant_h5	0.934	−15.9	35.44	−3.61	−2.35	27.4	23.68	(s.1pc) 11–17
ant_h6	0.948	−16.86	35	−1.41	−1.05	19.17	16.98	(s.1pc) 11–21
ant_h7	0.962	−17.67	35.56	0.74	−0.6	14.44	13.13	(s.2pc) 17–28
ant_h8	0.976	−19.09	34.14	−0.47	−0.38	11.51	10.66	(s.1pc) 17–30
ant_h9	0.988	−20.94	**33.8**	−0.34	0.29	10.01	9.37	(s.2pc) 27–36
								(c.1pc) 27–35
ant_h10	0.998	−22.24	**33.48**	−0.28	−0.24	9.01	8.54	(s.2pc) 27–36
								(c.1pc) 27–36
ant_h11	1.01	−24.35	**33.17**	−0.22	−0.2	8.17	7.76	(s.2pc) 27–36
								(c.1pc) 23–30
ant_h12	1.02	−26.44	**33**	−0.21	−0.19	7.86	7.49	(s.2pc) 24–36
								(c.1pc) 20–23

## Data Availability

Data sharing not applicable.
